# Care strategies for pregnant people and women living with hepatitis C in North America

**DOI:** 10.1016/j.xagr.2025.100580

**Published:** 2025-10-21

**Authors:** Sofia R. Bartlett, Jasmin E. Charles, Tatyana Kushner

**Affiliations:** 1 British Columbia Centre for Disease Control, Vancouver, British Columbia, Canada (Bartlett); 2 School of Population and Public Health, University of British Columbia, Vancouver, British Columbia, Canada (Bartlett); 3 Department of Obstetrics & Gynecology, University of Utah School of Medicine, Salt Lake City, UT (Charles); 4 Department of Internal Medicine, Program for Addiction Research, Clinical Care, Knowledge and Advocacy (PARCKA), Division of Epidemiology, University of Utah School of Medicine, Salt Lake City, UT (Charles); 5 Department of Gastroenterology and Hepatology, Department of Obstetrics and Gynecology, Weill Cornell Medicine, New York, NY (Kushner)

**Keywords:** case study, HCV screening, HCV, mother-to-child transmission, people who inject drugs, pregnancy, vertical transmission, viral hepatitis

## Abstract

Hepatitis C is a global health concern, with over 50 million people infected. Marginalized populations, particularly people who inject drugs, may not receive treatment despite an increased infection rate; similarly, there are gender disparities in the hepatitis C cascade of care, leaving some women undertreated. This is especially problematic, as hepatitis C rates are increasing substantially among individuals of childbearing age and pregnant people. While hepatitis C epidemiology and baseline characteristics are well understood, models of care focused on pregnant people that provide solutions to these barriers in attaining care are needed to increase health equity and achieve hepatitis C elimination. The adoption of highly effective, direct-acting antivirals for hepatitis C treatment has helped tremendously, but direct-acting antivirals must be accessible, and their availability must be combined with enhanced screening efforts. We review newly developed models of care for pregnant people who have hepatitis C and provide several case studies (with patient examples) of methods that have improved the care cascade and patient outcomes in our practices. Some models, such as mother-infant collocated care, allow postpartum and infant hepatitis C care to occur simultaneously, minimizing the number of visits and maximizing access to patient care. Other models, such as mobile point-of-care services and peer navigation, help marginalized populations attain access to care regardless of insurance status and transportation accessibility and provide peer support to help overcome treatment barriers, such as stigma and poverty. Additional innovative hepatitis C care models for pregnant people and women include modeling-based response-guided treatment, interdisciplinary collocated care models, and an integrated medical home model. Ultimately, there is no “one size fits all” hepatitis C model of care, as needs differ according to region, population demographics, and individual circumstances. As our review shows, many of the models apply multidisciplinary approaches to provide a range of care options. Reviewing the available models of care will help identify how practitioners can increase patient engagement with care and improve treatment uptake and completion rates among pregnant people and women and thus can contribute to hepatitis C elimination.

**AJOG Global Reports at a Glance**

**Why was this study conducted?**

Hepatitis C rates are increasing among individuals of childbearing age and pregnant people, but models of care for pregnant and postpartum populations require improvement to overcome barriers to screening and treatment

**What are the key findings?**

Mother-infant collocated care, peer navigation, mobile point-of-care services, and other multidisciplinary approaches have been shown to help improve access to hepatitis C testing and treatment for pregnant and postpartum populations.

**What does this study add to what is already known?**

There is no “one size fits all” model of care for hepatitis C, and we review multidisciplinary options that can be adapted in different settings to overcome barriers to access for pregnant and postpartum people.

## Introduction

Hepatitis C virus (HCV) is a significant global health concern. Chronic HCV infection is an important risk factor for the development of cirrhosis and hepatocellular carcinoma,^1^ and it is associated with extrahepatic manifestations, such as diabetes and renal disease.^2^, ^3^, ^4^ Globally, approximately 50 million people are chronically infected with HCV.^5^ Within North America alone, an estimated 204,000 people in Canada^6^ and 2.5 million people in the US are living with HCV.^7^

Along with the World Health Organization’s goal of achieving HCV elimination by 2030, both Canada and the US have set national goals^8^ to reduce rates of HCV-related deaths and incidence of new infections.^9^^,^^10^ Implementing interventions to enhance screening, linkage to care (LTC), and treatment of pregnant people and women is integral to achieving HCV elimination targets. Women face many barriers to HCV care, and if these are not specifically addressed, they may jeopardize elimination efforts and drive health inequities. Models of care that provide solutions to these barriers are urgently needed.

Here, we review models of care for women in North America in the direct-acting antiviral (DAA) era and provide case examples of methods that have improved the care process and patient outcomes.

### HCV in pregnant people and women

There are gender differences within the HCV cascade of care (COC), especially among higher-risk groups, such as people who inject drugs (PWID).^11^, ^12^, ^13^ Among women in Canada, the HCV prevalence rate in 2019 was 23.2 per 100,000; among women aged 25–29 years, the rate of HCV infection was even higher—32.3 per 100,000 people.^14^ In the US, from 2018–2022, the prevalence of acute HCV among women was 1.0 per 100,000 people.^15^ Rates of infection with HCV have been increasing among pregnant people in the US, with a 16-fold increase from 1998 to 2018,^16^ and among PWID, the rate of HCV is higher among women.^17^ The higher rate of HCV among women who inject drugs may stem from experiences of marginalization, resulting in a greater likelihood of engaging in higher-risk behaviors, such as borrowing used syringes and having sex partners who are also injecting.^18^^,^^19^

Treatment of HCV in people with childbearing potential is critical, because maternal HCV viremia can result in mother-to-child transmission (MTCT) of the virus, which may occur in up to 14% of pregnant people coinfected with HCV and HIV.^20^, ^21^, ^22^ Among women with HCV who are negative for HIV, the risk of vertical HCV transmission is approximately 5.8%.^22^ From 2011 to 2016, though pediatric testing for HCV remained low, it increased by 25%,^23^^,^^24^ yet studies still show that only 30% of infants and children who are perinatally exposed to HCV are screened.^25^ Perinatally infected children subsequently develop cirrhosis earlier than those who acquire HCV as adolescents, so timely detection and management of childhood HCV is crucial to mitigate the risk of cirrhosis and mortality among children infected via MTCT.^23^^,^^26^

Development of DAAs revolutionized HCV treatment by yielding very high sustained virologic response (SVR) rates (undetectable HCV viral load 12 weeks after treatment) and improved tolerability compared with interferon therapy; current DAA combinations consistently demonstrate SVR rates >92% against HCV genotypes 1 to 6.^27^, ^28^, ^29^ However, estimates suggest that in Canada and the US respectively, 24% and 32% of people infected with HCV are unaware of their infection,^6^^,^^30^ and only about 1 in 3 insured individuals gets timely treatment.^31^ In particular, women with concurrent social and health conditions (such as PWID) face many barriers to treatment, such as stigma, discrimination, and poverty,^32^^,^^33^ and are less likely to progress through the recommended stages of care.^13^

For persons who test positive for HCV during pregnancy, most national and international guidelines recommend waiting to start treatment postpartum,^34^^,^^35^ as data on DAA treatment during pregnancy are sparse (of note, there are 2 ongoing clinical trials for the treatment of HCV during pregnancy).^36^^,^^37^ However, the American Association for the Study of Liver Diseases guidelines specify that DAA treatment should be considered for pregnant individuals after risk analysis is performed, only on a case-by-case basis.^38^ Importantly, the Society for Maternal-Fetal Medicine recommends that DAA regimens be initiated during pregnancy only in clinical trial settings.^35^

Despite guideline recommendations, rates of initiation of HCV care following delivery among pregnant people are low. In a single US center, only 9% of pregnant people who tested positive for HCV initiated treatment within 1 year of delivery.^39^ A second US center reported that among people who were viremic during pregnancy, 17% (28 of 164) initiated DAA treatment within 15 months following delivery,^40^ and a third US center reported that less than 2% (6 of 369) of pregnant people with opioid use disorder (OUD) initiated treatment within 1 year of delivery.^41^

### HCV cascade of care

The COC is a valuable resource in the evaluation of various stages of the clinical care pathway for a specific illness.^42^ The HCV COC involves prevention efforts, screening, diagnosis, LTC, treatment, cure, and chronic care.^9^

### Individual strategies to improve HCV outcomes

#### Universal screening in pregnancy

Prenatal care is a critical opportunity to screen women for HCV. In recent years, HCV incidence in people of childbearing age has risen globally,^43^, ^44^, ^45^, ^46^ and HCV can be vertically transmitted from mother to infant.^47^ Screening during prenatal care and identifying those who are infected with HCV affords the opportunities to treat the mother as early as possible and to identify infections in neonates.^43^

Historically, healthcare systems in Canada and the US used risk-based models for prenatal screening.^48^, ^49^, ^50^ Risk factors included intravenous drug use (past or present), incarceration, hemodialysis, HIV-positive status, receipt of blood products before 1992, and tattoos or body modifications. Other risk factors included healthcare without universal precautions, needlestick injuries, birth to an HCV-positive mother, high-risk sexual activities/sex work, blood contact/shared care items with an HCV-positive individual, and elevated aminotransferase levels.^43^^,^^48^^,^^49^ Studies show that risk-based screening is largely ineffective.^51^^,^^52^ As such, the 2023 guidelines from the American College of Obstetricians and Gynecologists recommend universal screening for HCV during each pregnancy regardless of previous testing and results.^53^

Universal screening improves outcomes. One study found an 88% absolute increase in HCV screening among universal vs risk-based screened groups (99.9% vs 11.9%, respectively) of pregnant women. A higher prenatal HCV prevalence was identified through universal vs risk-based screening (0.11% vs 0.07%), and a greater number of people who participated in high-risk sexual activities were diagnosed with HCV through universal screening vs risk-based screening.^43^ This suggests that risk-based screening may overlook some high-risk patients.^43^ While universal screening identifies more women with HCV vs risk-based screening, HCV RNA reflex testing, or direct testing from HCV antibody–positive samples, is needed to ensure chronic infections are diagnosed promptly; studies have shown people are more likely to be cured if HCV RNA molecular testing is done within 6 months of serology.^54^, ^55^, ^56^

Despite HCV screening rates increasing in recent years, they still fall well short of universal recommendations. A retrospective study of patients with obstetric panels ordered showed 40.6% of pregnant Americans in quarter (Q) 2 of 2021 were screened compared with 16.6% in Q1 of 2011.^57^ In Canada, rates of HCV screening during pregnancy were 54.6% in 2019 compared to 19.6% in 2008^50^; of note, risk-based, rather than universal, screening is still the standard in Canada.^43^^,^^58^ For these reasons, it would also be helpful for HCV screening to be performed in all clinical settings where sexually transmitted infection (STI) screening may occur (eg, primary care physicians, STI screening clinics, family planning visits). It is of paramount importance that these gaps in screening practices are addressed if efforts toward HCV elimination are to succeed.

#### Peer navigation

Previous programs aimed at increasing engagement with HCV treatment and care have highlighted the role of social support.^59^^,^^60^ Peer navigation and/or social network interventions may help overcome barriers to HCV care, such as stigma or previous experiences of discrimination in healthcare settings.^33^^,^^61^ In a quality improvement project (QIP) in Canada, colocalized, peer-led testing and HCV treatment at a residential treatment center for women, combined with persistent follow-up efforts, led to increases in LTC and treatment: 5 of 13 women who were HCV RNA–positive initiated treatment on-site, while an additional 6 initiated treatment after leaving the center (Table).^62^

**Table:** Summary of HCV models of care in pregnant people or women with HCV

Model	Description	Example from published work and/or case studies
Peer navigation	Provides social support and/or social network interventions to overcome barriers to HCV care^59^, ^60^, ^61^, ^62^	QIP provided colocalized peer-led testing and HCV treatment at an addiction treatment center for women; it led to increased linkage in care and treatment^62^
Mobile point-of-care services	Service that allows marginalized women to receive HCV-related health services “where they are” regardless of health insurance or identification^63^	The CORNER Project targets sex workers to allow marginalized individuals access to specialized healthcare^63^
Mother-infant collocated care	Linkage program to provide collocated postpartum maternal and infant care to minimize appointment numbers and maximize follow-up care^40^	The SOFAR program was designed to support families affected by substance use; it successfully decreased time to treatment after delivery and increased HCV treatment rates^40^
Modeling-based RGT	Mathematical model-based approach to help shorten DAA treatment duration without compromising efficacy, which is especially useful in pregnant and postpartum women^64^	A retrospective study showed that RGT modeling allowed investigators to measure HCV on days 0, 7, and 14 of therapy (without days 2 and 28 of treatment), shortening therapy duration^64^^,^^82^
Integrated medical home model	MOUD program (methadone or buprenorphine) to help women with OUDs by decreasing opioid use and providing access to sterile needles to prevent HCV infection^66^^,^^70^	A pilot study in which 10 women in an outpatient MOUD program with OUD were given SOF/VEL; women had minimal side effects, and patient acceptability was high among women who received treatment^66^^,^^70^

*CORNER*, Washington Heights Corner Project; *DAA*, direct-acting antiviral; *HCV*, hepatitis C virus; *MOUD*, medication for opioid use disorder; *OUD*, opioid use disorder; *QIP*, quality improvement project; *RGT*, response-guided treatment; *SOFAR*, Supporting Our Families through Addiction and Recovery; *SOF/VEL*, sofosbuvir/velpatasvir.

#### Mobile point-of-care services

Due to homelessness and/or social and financial marginalization, access to specialists is a barrier to HCV care. The Washington Heights Corner Project (CORNER Project), established in 2005, was a peer-led, nurse-supported, community-based, mobile point-of-care service that targeted PWID and individuals who engaged in sex work in New York City and New Jersey.^63^ The CORNER Project used a mobile van to provide health services to people by meeting them where they were, without requiring health insurance or identification for access^63^ (Table). As of 2017, the CORNER Project (now OnPoint NYC) had helped 94 men and women secure access to HCV treatment; OnPoint NYC continues to advocate for social justice and addresses adverse outcomes among PWID.^63^

#### Mother-infant collocated care

The Boston Medical Center initiated a multidisciplinary clinic, the Supporting Our Families through Addiction and Recovery (SOFAR) program, to serve families affected by drug use/addiction by collocating postpartum maternal and infant care.^40^ This program was designed to maximize follow-up for the entire family while reducing the number of appointments needing to be made for mothers and their newborns.^40^ People who delivered during a linkage intervention period had approximately 3 times the rate of HCV treatment initiation when exposed to collocated care vs those who delivered prior to intervention.^40^ Such postpartum multidisciplinary programs can increase HCV treatment-initiation rates and may provide a framework on which health systems can build prenatal HCV testing (Table).

#### Modeling-based response-guided treatment

Mathematical modeling is a method that can predict time to cure by reproducing the biphasic viral decline on DAA therapy. This approach may be used to inform shortening of DAA duration without compromising treatment efficacy.^64^ Women indicated an interest in HCV treatment during pregnancy (to decrease the chance of vertical transmission), but some were hesitant to get such treatment due to a lack of safety data^65^; response-guided therapies may offer more individualized approaches that decrease medication exposure time during pregnancy (Table).

#### Integrated medical home model

HCV “cure as prevention” can be characterized by the use of medication for OUD (MOUD; eg, methadone, buprenorphine) and sterile syringe availability at needle/syringe programs and pharmacies, along with HCV treatment in one location.^66^ MOUD decreases HCV risk by reducing substance use^66^; studies show that MOUD reduces HCV infection by 40%–60%.^66^, ^67^, ^68^, ^69^ Investigators conducted a pilot study of sofosbuvir/velpatasvir among 10 pregnant/postpartum women with OUD in an outpatient program to evaluate the feasibility and acceptability of an OUD and HCV treatment model.^70^ At the time of publication, 6 women had initiated treatment postpartum: 3 completed treatment (2 confirmed SVR, 1 SVR result pending), 2 were still on treatment, and 1 discontinued due to substance use issues. The women reported minimal side effects and were satisfied with their treatment experience.^70^ While postpartum HCV treatment among women with OUD is challenging, this model shows promise as a way to initiate and sustain HCV treatment (Table).

### Case studies

The following case studies with specific patient examples provide some insight into real-world applications of novel HCV models of care.

**1: Substance Use and Pregnancy: Recovery, Addiction, Dependence clinic**

**Figure 1:**
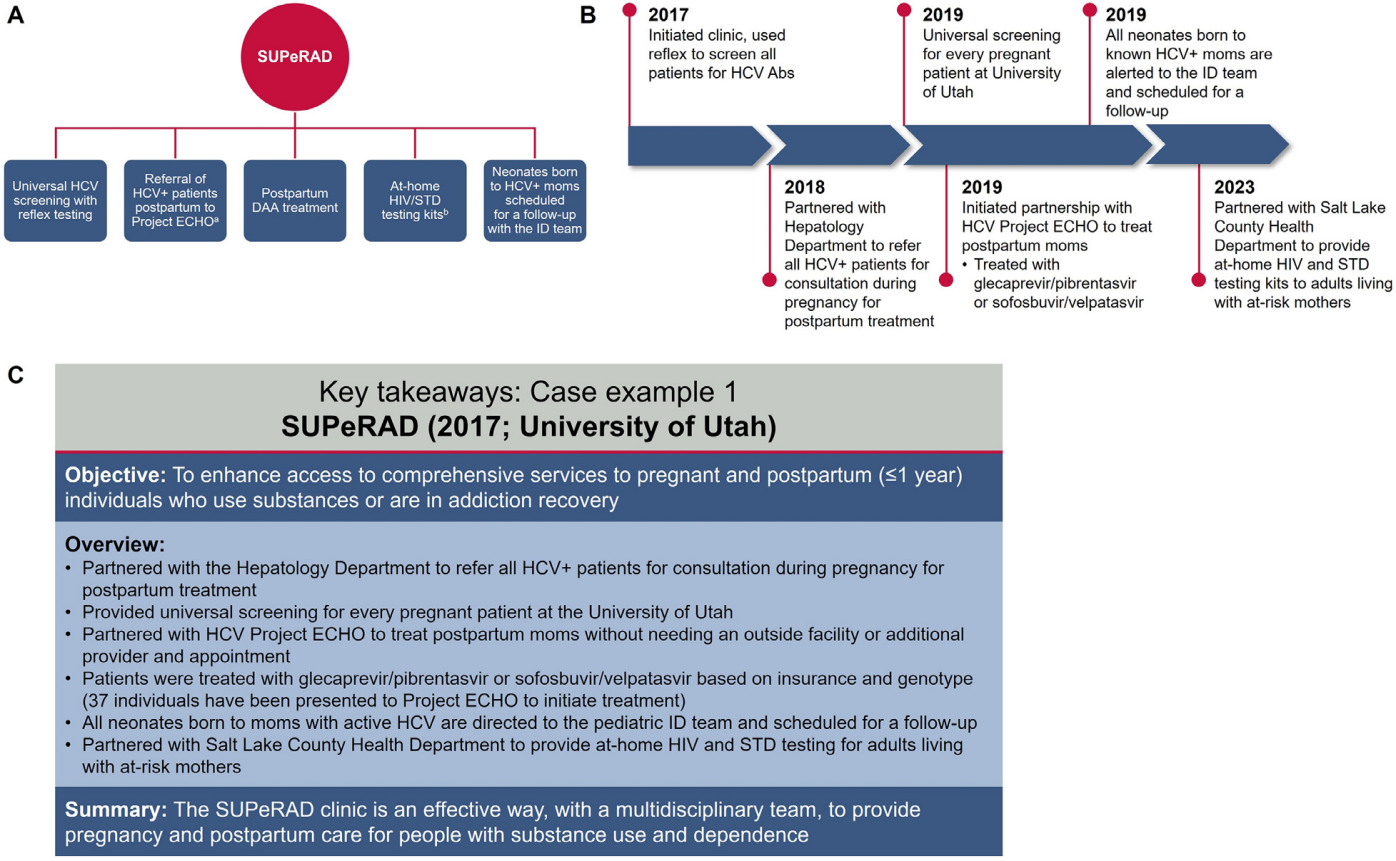
SUPeRAD prenatal specialty clinic: Summary of (A) key components of the program, (B) overview of the program development timeline, and (C) key takeaways. ^a^Patients who test positive for HCV can be referred to the Department of Hepatology only if they have a complicated case, such as a history of failed treatment or liver abnormalities. ^b^At-home HIV/STD testing kits are provided by the health department for partners/family members. *Ab*, antibody; *DAA*, direct-acting antiviral; *ECHO*, Extension for Community Healthcare Outcomes; *HCV*, hepatitis C virus; *ID*, infectious disease; *STD*, sexually transmitted disease; *SUPeRAD*, Substance Use and Pregnancy: Recovery, Addiction, Dependence.

The Substance Use and Pregnancy: Recovery, Addiction, Dependence (SUPeRAD) clinic, established in 2017 by members of the Department of Obstetrics and Gynecology (OBGYN) at the University of Utah, is a multi- and transdisciplinary perinatal addiction specialty clinic providing services for individuals who are pregnant or up to 1 year postpartum (Figure 1). The SUPeRAD clinic has partnered with multiple departments to provide universal HCV screening, postpartum DAA treatment, on-site peer support, at-home HIV and sexually transmitted disease testing kits for partners, and neonate follow-up, which work together to help diagnose and manage HCV in at-risk pregnant and postpartum women at a single location (Figure 1).

Bartlett. Care strategies for pregnant people and women living with hepatitis C in North America. AJOG Glob Rep 2025.

#### SUPeRAD patient 1

**Figure 2:**
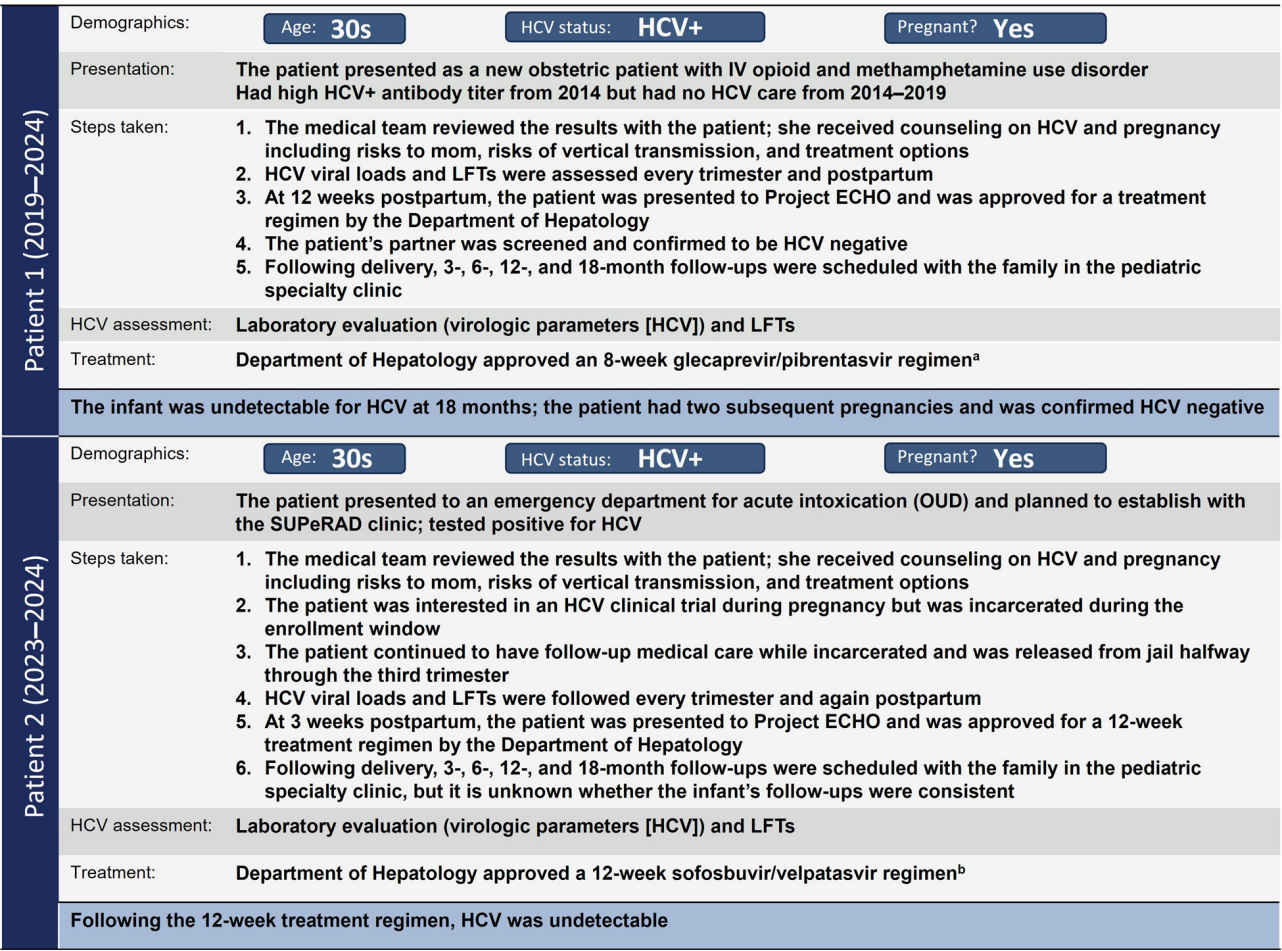
SUPeRAD patient examples. ^a^Medication was taken 3 times a day for 8 weeks. ^b^Medication was taken once daily for 12 weeks. *ECHO*, Extension for Community Healthcare Outcomes; *HCV*, hepatitis C virus; *IV*, intravenous; *LFT*, liver function test; *OUD*, opioid use disorder; *SUPeRAD*, Substance Use and Pregnancy: Recovery, Addiction, Dependence.

A pregnant woman in her 30s with active drug use presented as a new obstetric patient and screened positive for HCV. She received counseling on HCV and pregnancy, including risks to herself, risks of vertical transmission, and treatment options. HCV was assessed every trimester and again postpartum. At 12 weeks postpartum, the Department of Hepatology approved DAA treatment via Project Extension for Community Healthcare Outcomes (ECHO), a multidisciplinary training/mentoring model developed in New Mexico for primary care providers caring for patients with complex medical conditions in rural and underserved populations.^71^ HCV treatment postpartum is standard of care in the SUPeRAD clinic and includes completion of lab work, Project ECHO consultation for treatment approval, insurance approval, mail-ordered medication, and SVR at 12 weeks posttreatment. Following delivery, the infant was undetectable for HCV at 18 months. The patient had 2 subsequent pregnancies and was confirmed HCV negative (Figure 2).

Bartlett. Care strategies for pregnant people and women living with hepatitis C in North America. AJOG Glob Rep 2025.

#### SUPeRAD patient 2

A pregnant woman in her 30s presented with acute opioid intoxication. The patient tested HCV positive and received counseling for HCV and pregnancy. She was interested in an HCV clinical trial during pregnancy but was incarcerated during the enrollment window. The patient continued with follow-up medical care while incarcerated and was released from incarceration halfway through the third trimester. HCV viral loads and liver function tests were conducted every trimester and postpartum. At 3 weeks postpartum, the patient completed DAA/treatment approval through Project ECHO. HCV was undetectable at the end of treatment. Following delivery, the family was scheduled for follow-ups in the pediatric specialty clinic, but the status of the infant’s follow-ups is unknown (Figure 2).

**2: Women's Liver Clinic**

**Figure 3:**
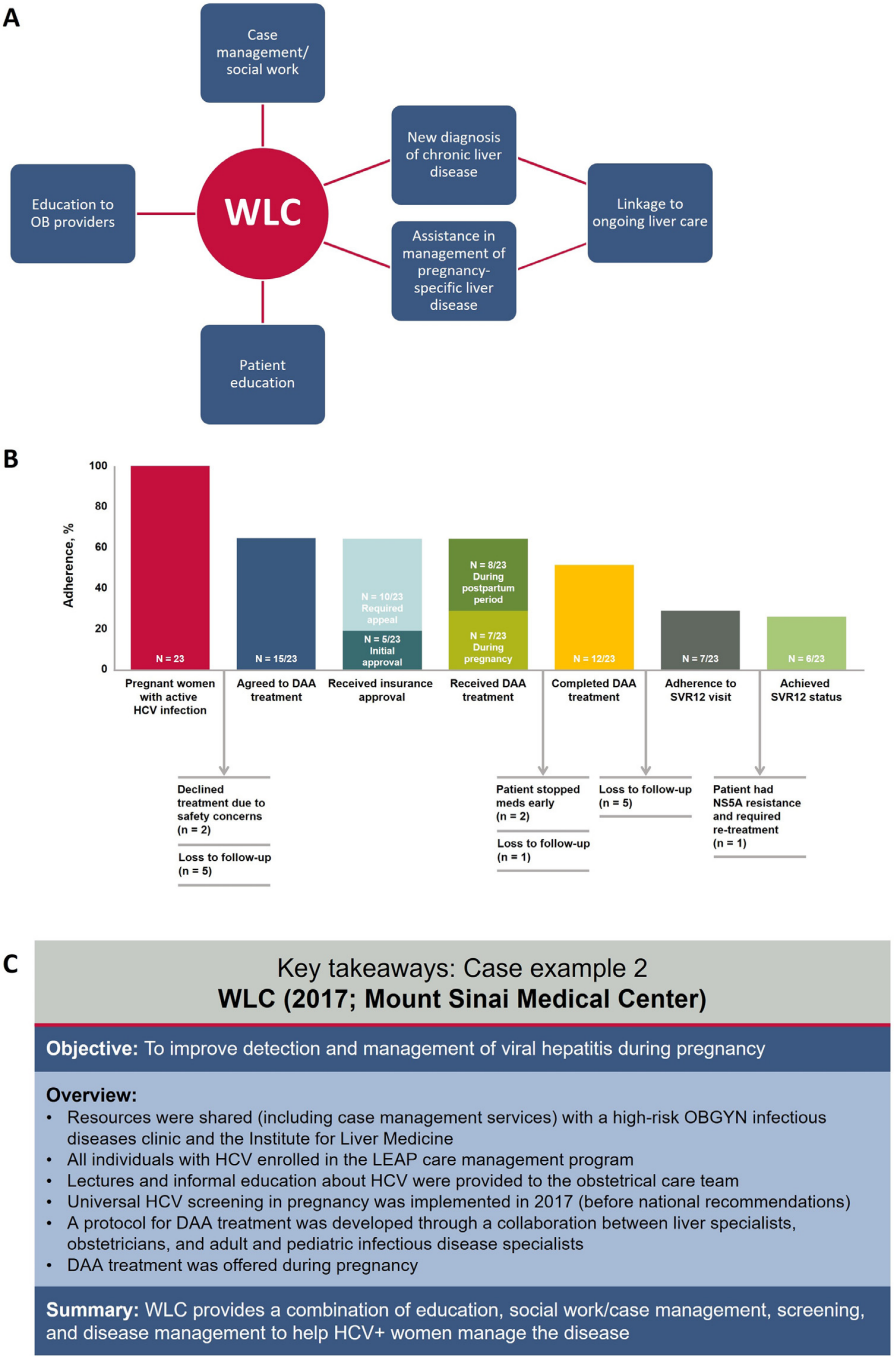
WLC: Summary of (A) key components of the program, (B) compliance with steps in the HCV pregnancy cascade of care, and (C) key takeaways. *Note:* Panel B is modified from Kushner T, et al. *Gastroenterology.* (2022), with permission. *DAA*, direct-acting antiviral; *HCV*, hepatitis C virus; *LEAP*, Liver Education and Action Program; *NS5A*, nonstructural protein 5A; *OB*, obstetrics; *OBGYN*, obstetrics and gynecology; *SVR12*, sustained virologic response 12 weeks posttreatment; *WLC*, Women’s Liver Clinic.

A weekly Women's Liver Clinic (WLC) was established in 2017 within the OBGYN ambulatory clinic practice at Mount Sinai Medical Center, New York, NY.^72^ The WLC shared resources with a high-risk OBGYN infectious disease clinic as well as with the Institute for Liver Medicine. All individuals with HCV were enrolled in the Liver Education and Action Program, a care coordination program, and institutional support was granted to implement universal HCV screening during pregnancy in 2017. Obstetric providers referred patients who had screened positive for HCV in pregnancy or who had known chronic HCV to the WLC. A protocol for DAA treatment was developed through collaboration between liver specialists, obstetricians, and adult and pediatric infectious disease specialists. DAA treatment was offered to women during pregnancy after discussion of available data on HCV treatment in pregnancy, and decisions to proceed with treatment were made jointly by patients, the OBGYN team, and the liver specialist (Figure 3).

Bartlett. Care strategies for pregnant people and women living with hepatitis C in North America. AJOG Glob Rep 2025.

#### WLC patient 1

**Figure 4:**
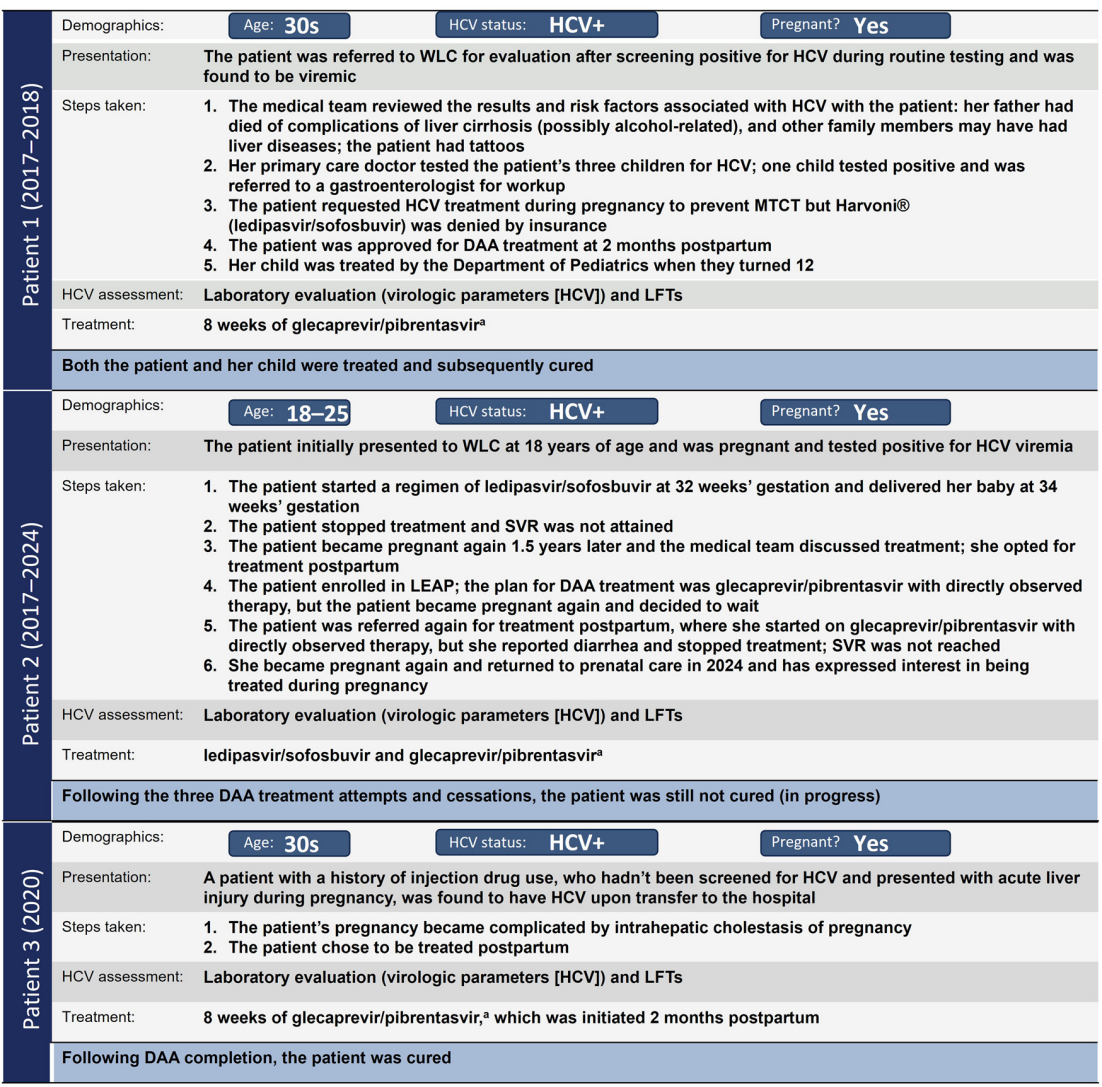
WLC patient examples. ^a^Medication was taken 3 times a day for 8 weeks. *DAA*, direct-acting antiviral; *HCV*, hepatitis C virus; *LEAP*, Liver Education and Action Program; *LFT*, liver function test; *MTCT*, mother-to-child transmission; *SVR*, sustained virologic response; *WLC*, Women's Liver Clinic.

A patient in her 30s visited the clinic after screening positive for HCV during routine pregnancy screening. On testing her children, 1 child tested positive. Both were treated and subsequently cured. The mother was treated postpartum (Figure 4).

Bartlett. Care strategies for pregnant people and women living with hepatitis C in North America. AJOG Glob Rep 2025.

#### WLC patient 2

A patient aged 18–25 years with HIV/HCV who had vertically acquired HCV was referred due to HCV-positive testing during pregnancy. After a discussion with the patient, the decision was made to initiate DAA treatment during pregnancy. The patient discontinued DAA treatment immediately after delivery after only completing approximately 2 weeks (delivered preterm). Treatment was subsequently reattempted in the postpartum period of a subsequent pregnancy, but cure was not achieved. The patient is pregnant again and considering HCV treatment during pregnancy (Figure 4).

#### WLC patient 3

A patient in her 30s with a history of injection drug use, who had not been screened for HCV and presented with acute liver injury during pregnancy, was found to have HCV upon transfer to the hospital. Pregnancy was further complicated by intrahepatic cholestasis of pregnancy. The patient chose to be treated postpartum and was cured of HCV infection postpartum (Figure 4).

**3: Test Link Call program**

**Figure 5:**
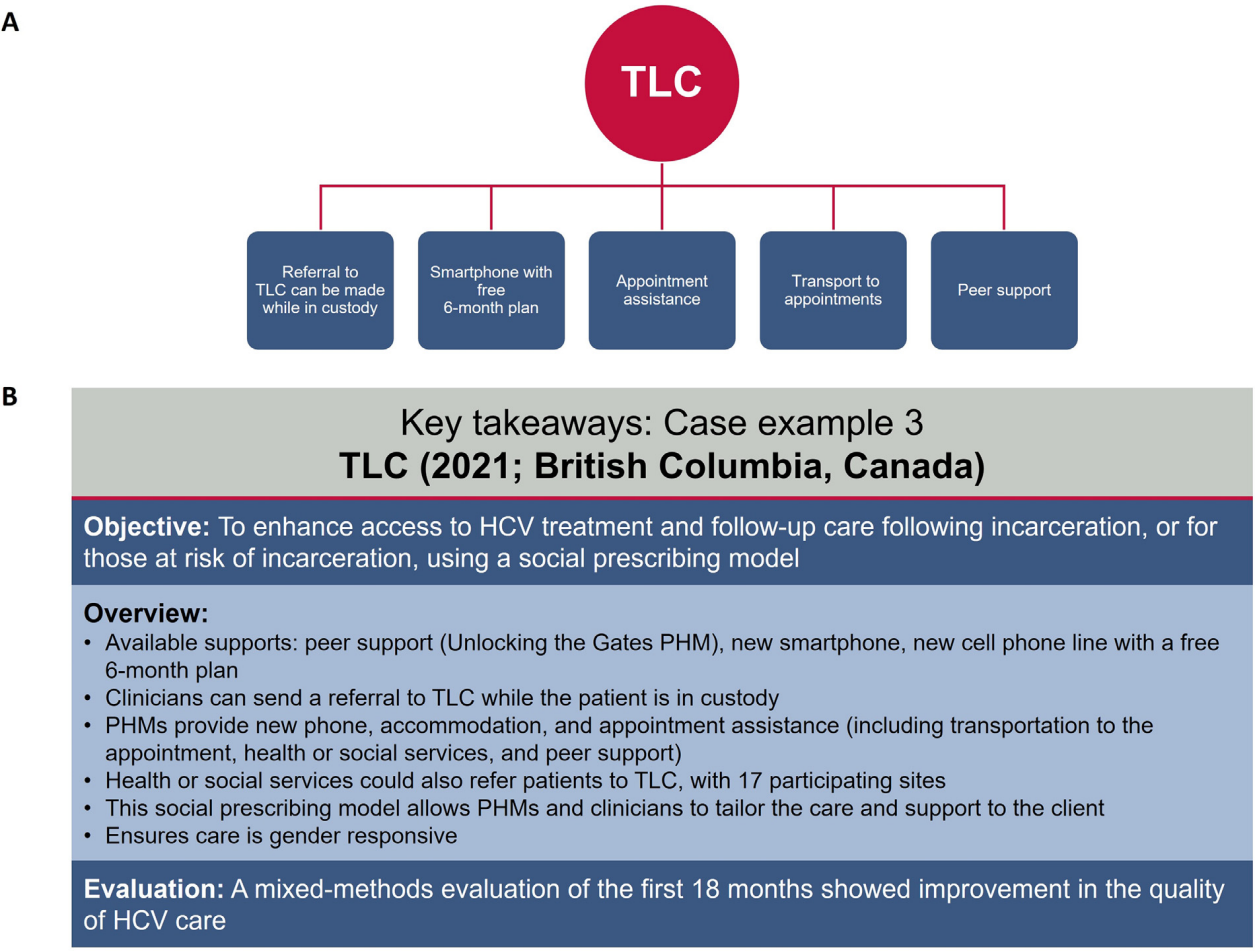
TLC: Summary of (A) key components of the program and (B) key takeaways. *HCV*, hepatitis C virus; *PHM*, peer health mentor; *TLC*, Test Link Call.

Test Link Call (TLC) is a QIP launched in 2021 in British Columbia, Canada.^73^ The objective of TLC is to enhance access to HCV treatment and follow-up care for people who are released from or at risk of incarceration. TLC provides tools to women, including peer support, new smartphones, and appointment assistance,^74^, ^75^, ^76^ to help ensure gender equity and responsiveness. A mixed-methods evaluation of the first 15 months showed improvement in quality of HCV care^77^ (Figure 5).

Bartlett. Care strategies for pregnant people and women living with hepatitis C in North America. AJOG Glob Rep 2025.

#### TLC patient 1

**Figure 6:**
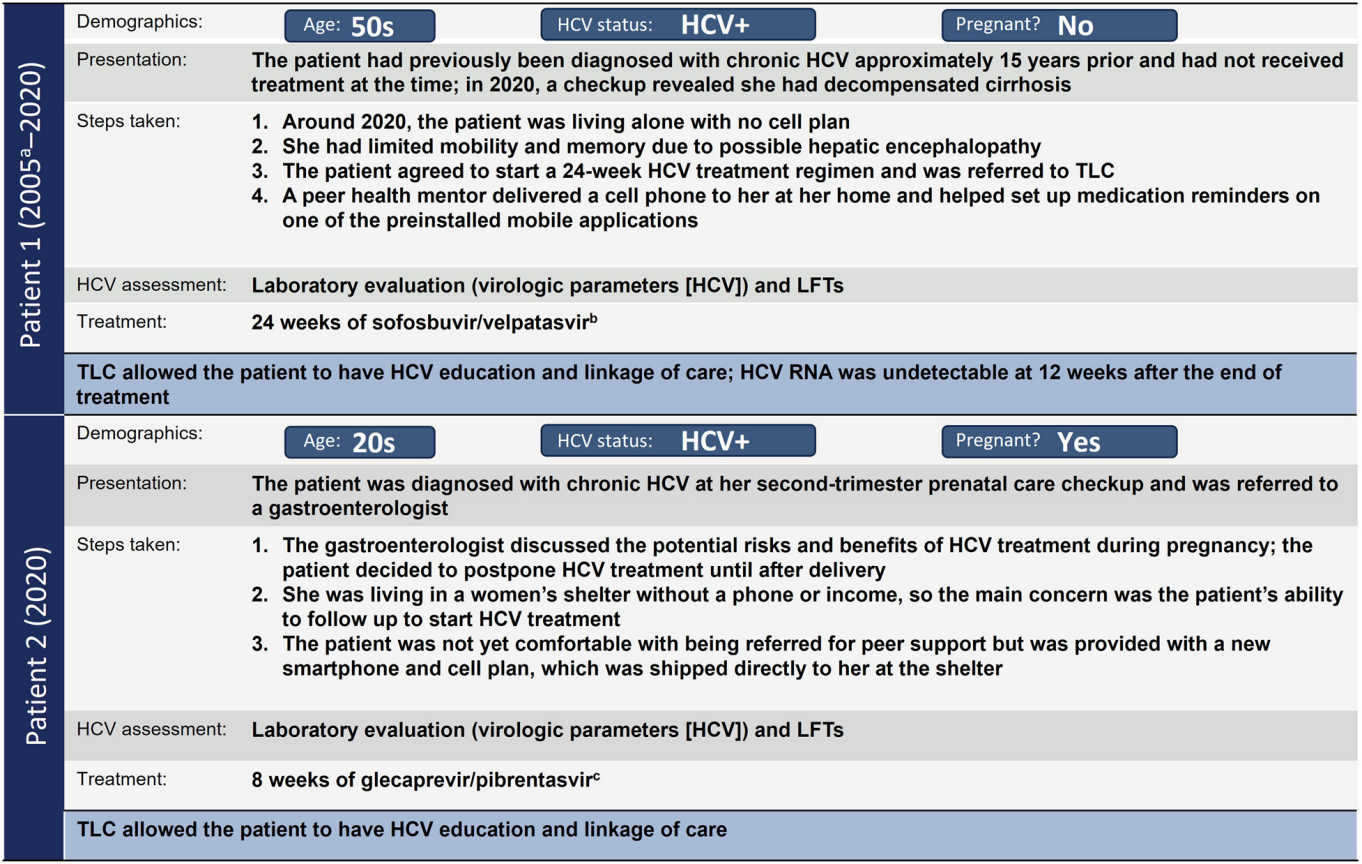
TLC patient examples. ^a^Patient was diagnosed with decompensated cirrhosis in 2020 but had been diagnosed with chronic HCV in 2005. ^b^Medication was taken once daily for 24 weeks. ^c^Medication administration began at 6 months postpartum and was taken 3 times a day for 8 weeks. *HCV*, hepatitis C virus; *LFT*, liver function test; *TLC*, Test Link Call.

A female patient in her 50s was diagnosed with chronic HCV approximately 15 years ago and had not received HCV treatment. In 2020, a checkup revealed she had decompensated cirrhosis. She had no cell phone plan and limited mobility and memory due to possible hepatic encephalopathy. The patient agreed to start HCV treatment and was referred to TLC. A peer health mentor delivered a cell phone to her with medication reminders on a mobile application. The patient successfully completed a 24-week DAA regimen, with HCV RNA undetectable at 12 weeks after the end of treatment (Figure 6).

Bartlett. Care strategies for pregnant people and women living with hepatitis C in North America. AJOG Glob Rep 2025.

#### TLC patient 2

A female patient in her 20s was diagnosed with chronic HCV at her second-trimester prenatal care checkup. She was referred to a gastroenterologist, who discussed the potential risks and benefits of HCV treatment during pregnancy. The patient decided to postpone HCV treatment until after delivery. The main concern was ensuring her ability to follow up to start HCV treatment without a phone or income. She was given a new smartphone, shipped directly to her at the women’s shelter where she was living, and a cell plan. She shared her phone number with her care providers, ensuring she was accessible for HCV treatment after delivery and to follow up with HCV screening for the infant at 18 months (Figure 6).

## Discussion

HCV is a major health concern; the number of cases of acute HCV has more than doubled since 2014 (129% increase).^78^ While research and understanding of epidemiology and baseline characteristics of HCV patients have improved, effective models of care are needed to improve delivery of HCV treatment. Decreasing barriers to obtaining care for pregnant people or women with HCV and increasing knowledge of potential models of care are essential to eliminating the disease.

A critical part of securing proper care for pregnant people with HCV is patient screening.^79^ Universal screening, ideally early during prenatal care, can help overcome the challenges of stigma and access. Additionally, when coupled with HCV RNA reflex testing, universal screening allows chronic infections to be diagnosed promptly.^43^^,^^54^

Social stigma and transportation inaccessibility due to marginalization are also barriers to HCV treatment. Peer navigation/networking and mobile point-of-care services are ways to help address these issues by meeting people where they are at. Mobile point-of-care services are linked to improved continuation of HCV care compared with standard HCV testing strategies.^80^ Mother-infant collocated care can provide simultaneous care, minimizing the number of appointments and increasing successful follow-up. Other programs, such as those using mathematical modeling to predict time to cure, could shorten DAA treatment time for pregnant and postpartum people, potentially increasing the likelihood of completing HCV treatment. Finally, the integrated medical home model was designed to help individuals with OUD by providing patients in an MOUD outpatient clinic with DAAs to help treat HCV and prevent further infection.

Among women who are freshly postpartum, there are several issues that may contribute to the suboptimal initiation of HCV treatment following delivery. Along with potential social/demographic issues that may complicate HCV treatment initiation, such as language barriers among migrant populations, there could be loss of healthcare insurance coverage postpartum, along with psychosocial stressors (eg, providing childcare to the new infant, needing to return to work), that could make it difficult to seek HCV treatment. As an example, in a study of 20 women with OUD in Kentucky, common barriers to treatment uptake included misinformation about treatment eligibility requirements (eg, lack of awareness of changes to insurance policies that now cover HCV treatment) and competing priorities during the postpartum period.^81^

## Conclusion

**Figure 7:**
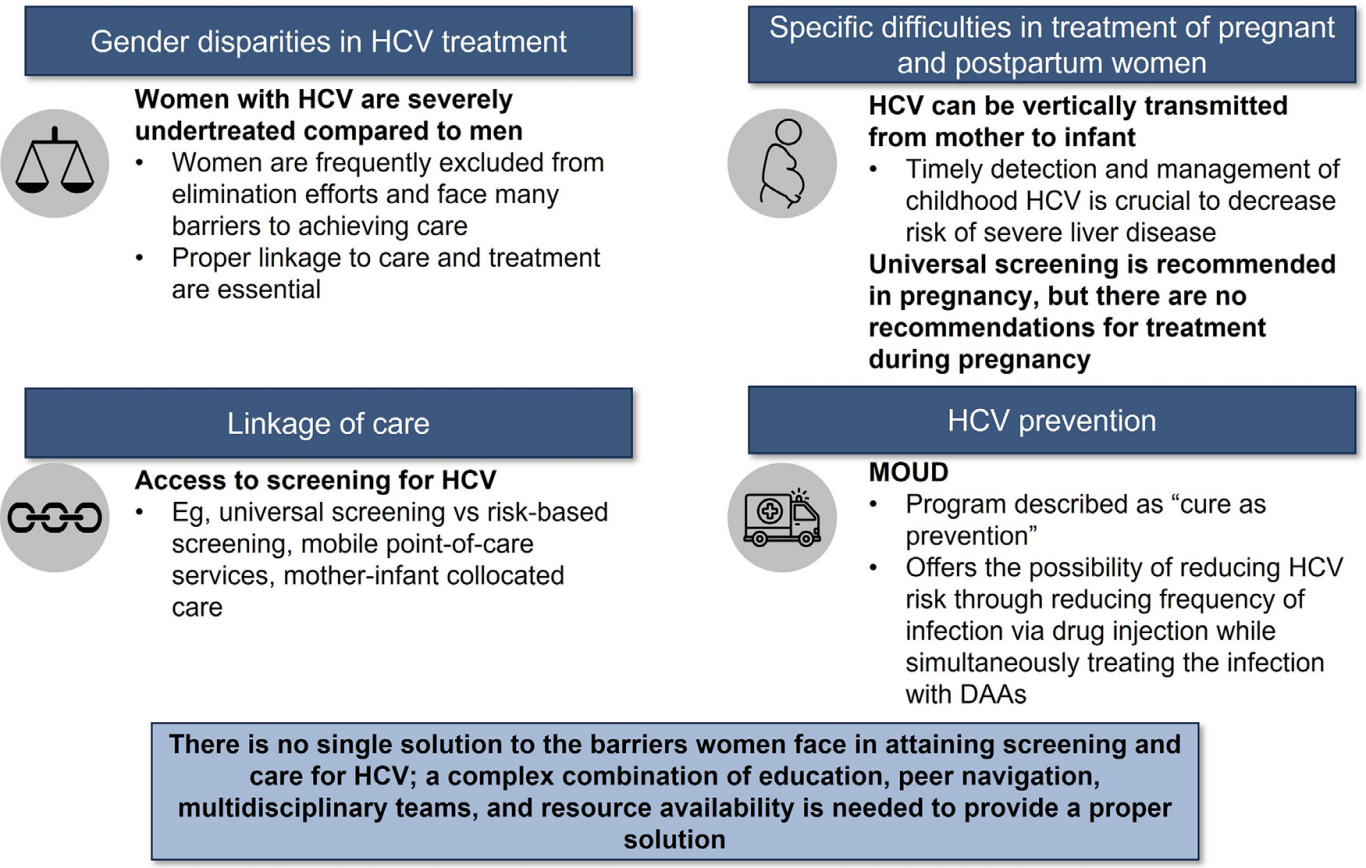
Key takeaways. *DAA*, direct-acting antiviral; *HCV*, hepatitis C virus; *MOUD*, medication for opioid use disorder.

In summary, several models have been developed in recent years to improve LTC and HCV treatment for pregnant people and women. The case studies we presented here use a combination of universal screening, mobile point-of-care services, and peer navigation, along with collocation of care and outreach. As crucial tools used by multidisciplinary teams, these care models assist people with HCV by providing LTC and treatment (Figure 7). Ultimately, implementing approaches identified in these different models can help improve access to HCV testing and treatment for pregnant and postpartum people.

Bartlett. Care strategies for pregnant people and women living with hepatitis C in North America. AJOG Glob Rep 2025.

## CRediT authorship contribution statement

**Sofia R. Bartlett:** Writing – review & editing, Validation, Data curation, Conceptualization. **Jasmin E. Charles:** Writing – review & editing, Validation, Data curation, Conceptualization. **Tatyana Kushner:** Writing – review & editing, Validation, Data curation, Conceptualization.
